# Impact of a digital pre- and rehabilitation program on postoperative outcomes after pelvic gynecological surgery

**DOI:** 10.3389/fdgth.2026.1867518

**Published:** 2026-06-26

**Authors:** Fabien Vidal, Guillaume Ploussard, Jean-Dominique Bernard, Justine Figurelli

**Affiliations:** 1 Department of Surgical Gynecology, La Croix du Sud Hôpital, Quint Fonsegrives, France; 2 Department of Urology, La Croix du Sud Hôpital, Quint Fonsegrives, France

**Keywords:** eHealth, gynecology, oncology, prehabilitation, remote monitoring, surgery

## Abstract

**Background:**

Growing evidence suggests that structured preoperative education and close postoperative monitoring improve surgical outcomes. Digital health solutions may facilitate the large-scale implementation of perioperative optimization pathways by integrating prehabilitation, patient education, and remote monitoring into routine care.

**Objective:**

To evaluate the impact of implementing a comprehensive digital perioperative coaching program (BETTY) on postoperative outcomes in patients undergoing elective pelvic gynecological surgery.

**Methods:**

This retrospective, single-center, pre- and postintervention study was conducted in a high-volume tertiary hospital. Consecutive adult patients undergoing elective pelvic gynecological surgery by a single experienced surgeon between February 2024 and January 2025 were included. During the fourth quarter of the study period, a digital perioperative pathway incorporating prehabilitation modules, structured education, rehabilitation guidance, and electronic patient-reported outcomes (ePROs) was implemented. The primary endpoint was the 90-day readmission rate. Secondary endpoints included length of stay (LOS) and high-grade postoperative complications. Outcomes before implementation (Q1–Q3) were compared with those after implementation (Q4).

**Results:**

A total of 426 patients were analyzed, including 313 in the preimplementation group and 113 in the postimplementation group. Baseline characteristics and surgical complexity were comparable between groups. The 90-day readmission rate decreased from 12.8% before implementation to 8.8% after implementation, corresponding to a 34% relative reduction (*p* = 0.17). High-grade complications decreased from 1.6% to 0.9% (*p* = 0.5). Overall length of stay remained stable (0.86 days), with a same-day discharge rate of 54.2%. Comparison with national data confirmed the more favourable post-operative profile in terms of same-day-discharge rate (*p* = 0.004) and readmission rate (*p* < 0.001).

**Conclusions:**

The implementation of a structured digital perioperative pathway integrating prehabilitation and remote monitoring was associated with lower readmission and complication rates after pelvic gynecological surgery. Although differences did not reach statistical significance, the magnitude of effect suggests potential clinical relevance and supports further prospective evaluation.

## Introduction

Pelvic gynecological surgery encompasses a broad spectrum of procedures performed for benign and complex conditions, including endometriosis, uterine fibroids, adnexal pathology, pelvic pain syndromes, and abnormal uterine bleeding. Although advances in minimally invasive techniques, anesthesia protocols, and enhanced recovery pathways have substantially reduced perioperative morbidity and shortened hospital stays, postoperative complications and unplanned readmissions remain clinically relevant challenges. Even in high-volume centers, early postoperative events such as pain exacerbation, bleeding, infection, urinary dysfunction, or gastrointestinal disturbances may lead to emergency consultations or rehospitalization (1–3). These events contribute to patient distress, healthcare system burden, and increased costs. Therefore, optimizing perioperative management beyond technical surgical performance remains a strategic priority (4).

Prehabilitation has emerged as a critical component of perioperative optimization (5, 6). Conceptually, prehabilitation aims to increase patients' physiological reserve before surgery through multimodal interventions targeting physical conditioning, respiratory function, nutritional status, and psychological preparedness. Meta-analyses in abdominal and oncologic surgery have demonstrated improvements in functional capacity and, in selected populations, reductions in postoperative complications (7, 8). Despite promising results, the implementation of structured prehabilitation programs in routine clinical practice remains inconsistent. Barriers include limited availability of specialized staff, logistical constraints, patient travel requirements, and scheduling complexity (9). As a result, access to prehabilitation is frequently restricted to tertiary centers or research settings (10).

In parallel, digital health technologies have transformed the delivery of healthcare services. Telemedecine may help to overcome financial and organizational constraints, and to spread perioperative pathways to a wider audience of patients (10, 11). Digital programs have proven to be at least as effective as on-site perioperative programs in prospective trials and may lead to improved recovery and satisfaction after major surgery (12–14). Smartphone penetration has reached high levels across adult populations in industrialized countries, creating an opportunity to deploy scalable, patient-centered digital interventions. Mobile health (mHealth) applications can deliver educational content, structured exercise programs, behavioral nudges, and real-time symptom monitoring with minimal incremental cost per patient. In the perioperative context, digital platforms may facilitate standardized education, reinforce adherence to recovery milestones, and enable remote monitoring through electronic patient-reported outcomes (ePROs) (15). Randomized trials have demonstrated that personalized eHealth programs can accelerate functional recovery and return to work after gynecological surgery, and that digital perioperative care models may reduce healthcare utilization after abdominal surgery (12, 16).

Gynecological surgery constitutes an appropriate setting for evaluating digital perioperative interventions. Many procedures are performed in relatively young, active patients who are accustomed to smartphone use. Moreover, a substantial proportion of surgeries are performed in ambulatory or short-stay settings, increasing reliance on patient self-management after discharge. Although minimally invasive techniques have reduced overall morbidity, symptoms such as pelvic pain, urinary discomfort, bowel dysfunction, or fatigue remain frequent during early recovery. Structured digital guidance may therefore support symptom normalization, improve adherence to mobilization and analgesic protocols, and reduce unnecessary healthcare contacts (8, 12).

Despite the theoretical advantages of digital perioperative pathways, real-world evidence in gynecological surgery remains limited. Most existing data originate from randomized trials conducted under controlled research conditions, often focusing on selected patient populations or specific outcomes such as return to work (12). Implementation studies assessing integration into routine high-volume surgical practice are scarce. Furthermore, the combined impact of integrating prehabilitation, education, rehabilitation, and remote monitoring within a single unified digital platform has not been extensively evaluated in pelvic gynecological procedures.

The BETTY digital coaching program was developed as a CE-marked medical device designed to deliver a comprehensive perioperative pathway via smartphone application. The platform integrates multimodal prehabilitation modules, structured educational content, daily postoperative guidance, and ePRO-based monitoring with clinician alerts. A prior prospective multicenter study in urologic surgery suggested that this model may reduce complications and improve recovery metrics (17, 18). Whether similar benefits extend to gynecological surgery in a real-world, nonrandomized implementation context has not been determined.

The present study was therefore designed to evaluate the impact of implementing this digital perioperative pathway in a high-volume tertiary center performing complex pelvic gynecological surgery. We hypothesized that systematic integration of digital prehabilitation and rehabilitation would be associated with a reduction in 90-day readmission rates and high-grade complications without adversely affecting length of stay. By comparing outcomes before and after implementation while maintaining stable surgical and perioperative protocols, this study aimed to isolate the potential contribution of the digital intervention within an established enhanced recovery framework.

Through this analysis, we sought to contribute implementation-level evidence to the evolving field of digital perioperative medicine and to assess whether scalable mobile health solutions can meaningfully complement established surgical optimization strategies in routine gynecological practice.

## Methods

### Study design and setting

This study was a retrospective, single-center, observational pre-post implementation cohort study conducted in a high-volume tertiary referral center specializing in complex pelvic gynecological surgery. Consecutive adult patients undergoing elective pelvic gynecological surgery for benign and oncological indications were included. Surgical indications comprised endometriosis, hysterectomy, adnexal pathology, and gynecological oncologic procedures. Institutional perioperative management followed standardized enhanced recovery after surgery (ERAS) principles throughout the study period.

The objective of the study was to evaluate whether implementation of a comprehensive digital perioperative pathway integrating structured education, prehabilitation guidance, rehabilitation, and electronic patient-reported outcomes (ePROs) was associated with changes in short-term postoperative outcomes. The study period extended from February 1, 2024, to January 31, 2025.

The study was conducted in accordance with the Declaration of Helsinki and Good Clinical Practice principles.

### Study population

All consecutive adult patients (≥18 years) undergoing elective pelvic gynecological surgery performed by a single experienced surgeon during the study period were eligible for inclusion. Restricting surgical procedures to a single surgeon minimized inter-operator variability and procedural heterogeneity.

Inclusion criteria were:

1. Age ≥18 years.
2. Elective pelvic gynecological surgery requiring general anesthesia.
3. Availability of complete perioperative and follow-up data for at least 90 days.

Exclusion criteria were:

1. Emergency surgical procedures.
2. Minor ambulatory diagnostic procedures without anesthesia.
3. Incomplete registry data.
4. Patients unable to use a smartphone device in the postintervention phase and declining caregiver support (no patient excluded for this reason).

No modifications were made to surgical techniques, anesthesia protocols, analgesic regimens, antibiotic prophylaxis, thromboprophylaxis, or discharge criteria during the study period. This methodological control ensured that the digital intervention was the sole structural change between the pre- and postimplementation cohorts. Consecutive inclusion was used to minimize selection bias. Outcome capture relied on the PMSI national registry, limiting loss to follow-up for hospitalization-related outcomes.

### Perioperative management and digital intervention

Perioperative surgical and anesthetic protocols remained intentionally stable throughout the study period in order to reduce temporal confounding and isolate the potential contribution of the digital intervention. No modifications were made to surgical techniques, anesthesia protocols, analgesic regimens, thromboprophylaxis, antibiotic prophylaxis, discharge criteria, or institutional ERAS pathways during the study period.

Before implementation, patients received standard perioperative care including surgical consultation, anesthesiology assessment, perioperative counseling, minimally invasive surgery whenever feasible, multimodal analgesia, and early mobilization/discharge protocols. Patient education relied primarily on face-to-face consultations and written or oral instructions, without structured digital monitoring or remote follow-up.

At the beginning of the fourth quarter of the study period (October 2024), the BETTY digital perioperative pathway (AIMED2, Toulouse, France) was implemented (17). BETTY is a CE-marked smartphone-based digital medical device designed to support perioperative care. All eligible patients during the postimplementation phase were invited to download and use the application. The principal functionalities of the patient and clinician interfaces have been previously described.

The BETTY pathway was integrated into routine ERAS care and included:

1. Structured educational content regarding surgery, recovery expectations, pain management, mobilization, and warning symptoms;
2. Prehabilitation guidance including physical activity promotion, respiratory exercises, diet, smoking-related recommendations, and behavioral advice;
3. Postoperative rehabilitation coaching with recovery milestones and discharge support;
4. ePRO.

The Table 1 summarizes the main contents.

**Table 1:** Content and educational material included in the BETTY coaching program.

Physical activity before surgery
Medical history form
Perioperative smoking
Breathing exercises
Dietary recommendations
Anesthesia techniques and checklist
Preoperative ePROs
Smokers: instructions to follow before your surgery
Pain management
Care pathway up to surgery
Admission checklist
Skin preparation
Care pathway from surgery to return home
Postoperative assessment form
Scar care: best hygiene practices
Postoperative recovery: key recommendations for the first days
Postoperative ePROs at 6 weeks, 6 months and 1 year
Postoperative follow-up checklist

The digital pathway was initiated following surgical scheduling and continued during postoperative recovery. No dedicated multidisciplinary prehabilitation clinic or formal frailty screening pathway was implemented during the study period. Preoperative evaluation remained supervised by the surgeon and anesthesiologist according to institutional routine practice. Nutritional support was limited to general perioperative dietary recommendations delivered through the application; no formal nutritional intervention or supplementation protocol was systematically implemented.

### Outcome measures

The primary endpoint was the 90-day readmission rate. Readmission was defined as any unplanned hospitalization through the emergency department or surgical ward occurring within 90 days after index surgery, regardless of duration.

Secondary endpoints included:

- Length of stay (LOS) during index hospitalization
- LOS during readmission
- High-grade postoperative complications
- Temporal stability of readmission rates by quarter

### Complication classification

High-grade complications were defined according to the institutional Severity Index (SI) algorithm derived from PMSI coding. The SI integrates comorbidities, complication codes, procedural complexity, and length of stay as follows: SI 1–2: uncomplicated cases; SI 3–4: complicated cases representing high-grade complications. A severity index (SI) score, encoded at hospital discharge, was also included. In the French health care system, patients are classified according to four levels of severity on the basis of risk factors, comorbidities, complications, and LOS. An algorithm determines the level of severity for each patient and provides the corresponding reimbursement rate for the procedure that the institution can claim. Levels SI1 and SI2 indicate uncomplicated cases, whereas SI3 and SI4 are associated with surgical complications in 85% of cases (19). Although Clavien–Dindo classification is more widely recognized internationally, SI-based assessment is routinely used within the French healthcare reimbursement system and was consistently available for all patients included in this registry-based analysis.

### Data source

All data were extracted from the French national hospital discharge database, Programme de Médicalisation des Systèmes d'Informations (PMSI). The PMSI registry collects standardized medico-administrative and diagnostic data for all hospital stays in France. Data extraction included demographic variables, procedure codes (CCAM classification), LOS, readmission events, and severity indices. Registry-based outcome assessment ensures completeness of hospitalization data and reduces reporting bias.

### Statistical analysis

Patients were divided into two groups:

- Preimplementation cohort (Q1–Q3)
- Postimplementation cohort (Q4)

Continuous variables were expressed as means ± standard deviation or medians with interquartile ranges depending on distribution normality. Categorical variables were expressed as frequencies and percentages. Comparisons between groups were performed as follows:

- Chi-square test or Fisher's exact test for categorical variables
- Mann–Whitney *U* test for continuous nonparametric variables
- Independent *t*-test for normally distributed continuous variables

Temporal trends across quarters were evaluated descriptively.

We also did a nationwide comparison of post-operative outcomes between all private practices centers in France and our department for oncologic surgery. Using the provided 2024 VISUCHIR comparative extract, we descriptively compared same-day discharge (SDD) rate, inpatient hospital stay (days), and the VISUCHIR-reported 5-year evolution of the 30-day readmission rate. Analyses were performed for all oncologic gynecology cases. This study used aggregated, publicly available benchmarking data and did not involve individual-level patient data. The National data (private practice centers only) were compared with data from four early-adopter private practice departments.

**Figure 1:**
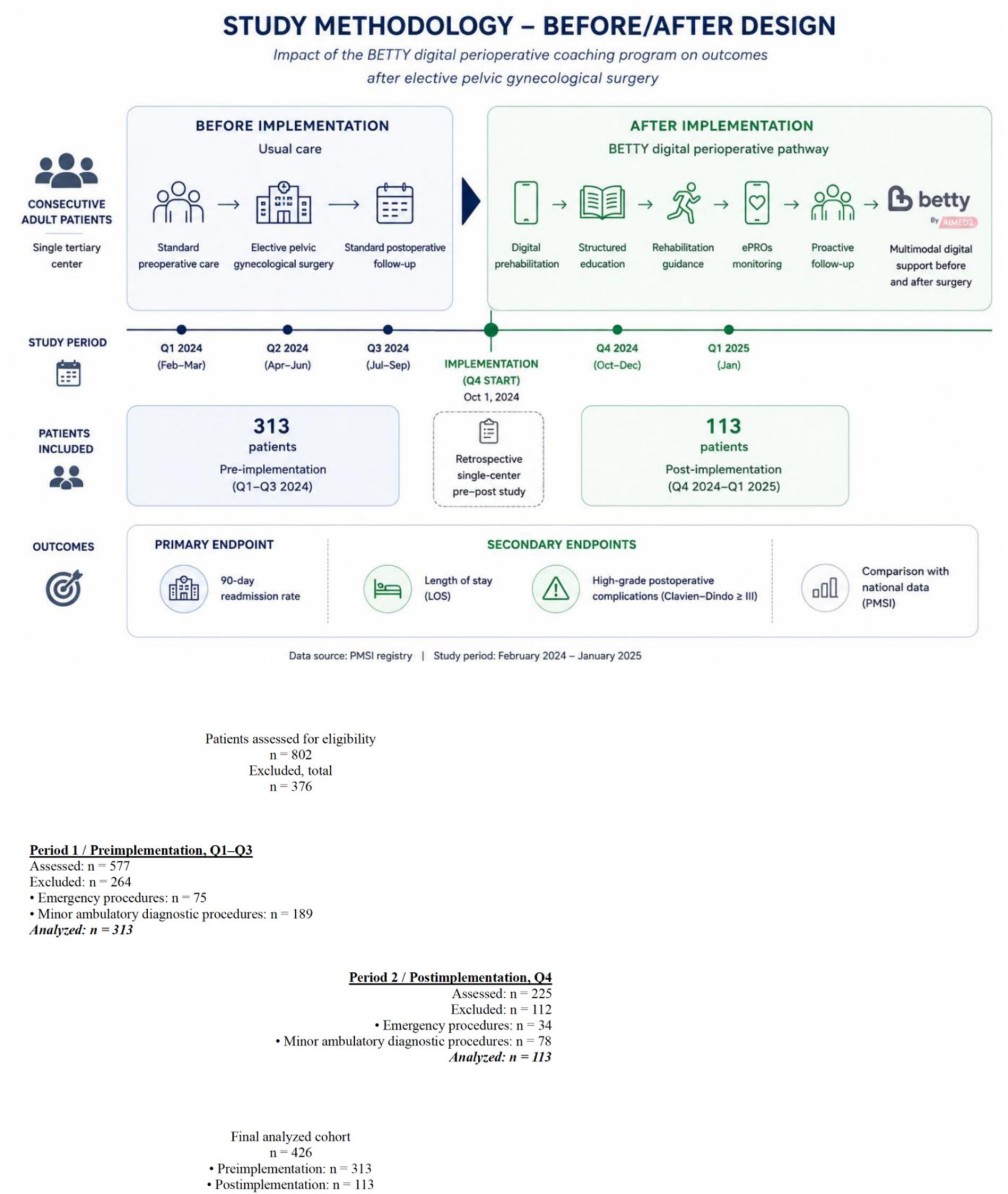
Study flow and design.

Flow chart of the study has been detailed in Figure 1.

All statistical analyses were performed using SPSS version 22.0 (IBM Corp., Chicago, IL). A two-sided *p*-value <0.05 was considered statistically significant. Given the exploratory nature of this pre-post implementation study, no *a priori* sample size calculation was performed.

## Results

A total of 426 patients were enrolled, including 313 (Q1–3) and 113 (Q4) before and after the implementation of the digital pathway. Patients' characteristics and outcomes are presented in Table 2.

**Table 2:** Patient characteristics and outcomes.

Characteristics and Outcomes	Overall cohort *n* = 426	Before *n* = 313	After *n* = 113	*p* value
Age, years	39.9	39.8	40.4	0.6
Number of surgical procedures per patient, *n* (%)	2.9	2.8	3.0	0.5
Main Surgery procedures, *n* (%)				0.9
Peritoneal endometriosis excision	155 (36.4)	109 (34.8)	46 (40.7)	
Rectovaginal endometriosis excision	122 (28.6)	87 (27.8)	35 (31.0)	
Hysterectomy	85 (20.0)	62 (19.8)	23 (20.4)	
Oophorectomy/Ovarian cystectomy	66 (15.5)	51 (16.4)	15 (13.3)	
Salpingectomy	136 (31.9)	98 (31.3)	38 (33.6)	
Ureterolysis	211 (49.5)	154 (49.2)	57 (50.4)	
Operative hysteroscopy	141 (33.1)	102 (32.6)	39 (34.5)	
Peritoneal adhesiolysis	63 (14.8)	45 (14.4)	18 (15.9)	
Laparotomy approach	14 (3.3)	10 (3.2)	4 (3.5)	
High-grade complications^a^, *n* (%)	6 (1.4)	5 (1.6)	1 (0.9)	0.5
95% CI	0.6–3.0	0.7–3.7	0.2–4.8	
LOS, days (mean)	0.86	0.87	0.85	0.9
Median, IQR	1 (0–1)	1 (0–1)	1 (0–1)	
LOS if readmission, days	2.8	3.2	2.1	0.3
Median, IQR	1 (1–2)	1 (1–3)	1 (1–2)	
Readmission, *n* (%)	50 (11.7)	40 (12.8)	10 (8.8)	0.17
95% CI	9.0–15.1	9.5–16.9	4.9–15.5	

LOS, length of stay.

a High grade complications were evaluated by the severity index which classified patients into 4 levels of severity, according to risk factors, comorbidities, complications and length of stay. An algorithm determines the level of severity for each patient and provides the corresponding reimbursement rate to be pursued by the institution for the procedure. Levels (severity index: SI) 1 or 2 indicate non-complicated cases whereas levels 3 and 4 are associated with surgical complications; CI, confidence interval; IQR, interquartile range.

Mean age was comparable before and after the implementation of the app (39.8 vs. 40.4 years, *p* = 0.6). The mean number of surgical procedures per patient was 2.8 and 3.0 before and after implementation (*p* = 0.5). Repartition of main surgery procedures did not differ significantly between groups (*p* = 0.9).

Baseline demographic characteristics and surgical complexity remained stable throughout the study period. Importantly, the surgical case mix did not significantly differ between periods (*p* = 0.9), supporting the comparability of the two cohorts. The distribution of major procedures remained balanced over time, including endometriosis excision procedures (peritoneal and rectovaginal), hysterectomy, oophorectomy or ovarian cystectomy, salpingectomy, ureterolysis, operative hysteroscopy, and peritoneal adhesiolysis. The proportion of laparotomy approaches also remained low and stable (3.2% before vs. 3.5% after implementation), confirming the consistency of surgical practice and procedural complexity across study phases. These findings suggest that differences observed in postoperative outcomes were unlikely to be explained by major variations in patient characteristics or operative case mix between the two periods.

Additional details regarding postoperative morbidity and hospitalization metrics were incorporated to better characterize the overall perioperative profile of the cohort. High-grade complications remained infrequent overall, reflecting the minimally invasive and ERAS-oriented surgical practice of the center. Nevertheless, severe complications decreased numerically from 1.6% before implementation to 0.9% after implementation. Length of stay during index hospitalization remained highly stable at approximately 1 day (median value), with more than half of patients managed in a same-day discharge setting. The 90-day readmission rate was reduced from 12.8% to 8.8% after the implementation (*p* = 0.17). An analysis by time quarter showed a stable rate during quarter 1–3 before the implementation (Q1: 12.4%; Q2: 14.0%; Q3: 12.0%). In case of readmission, the length of stay was longer by almost 1 day before the implementation (*p* = 0.3).

A multivariable analysis taking into account age, number of procedures, surgical approach and period (before/after implementation) is showed in Table 3.

**Table 3:** Multivariable analysis taking into account age, number of procedures, surgical approach, and period (before versus after implementation) for 90-day readmission rate prediction.

Variable	*P* value	OR	95%CI
Age	0.103	1.02	0.99–1.05
Number of surgical procedures	0.719	0.86	0.38–1.92
Digital pathway implementation	0.309	0.67	0.32–1.43
Laparoscopy	0.727	0.69	0.09–5.5

**Figure 2:**
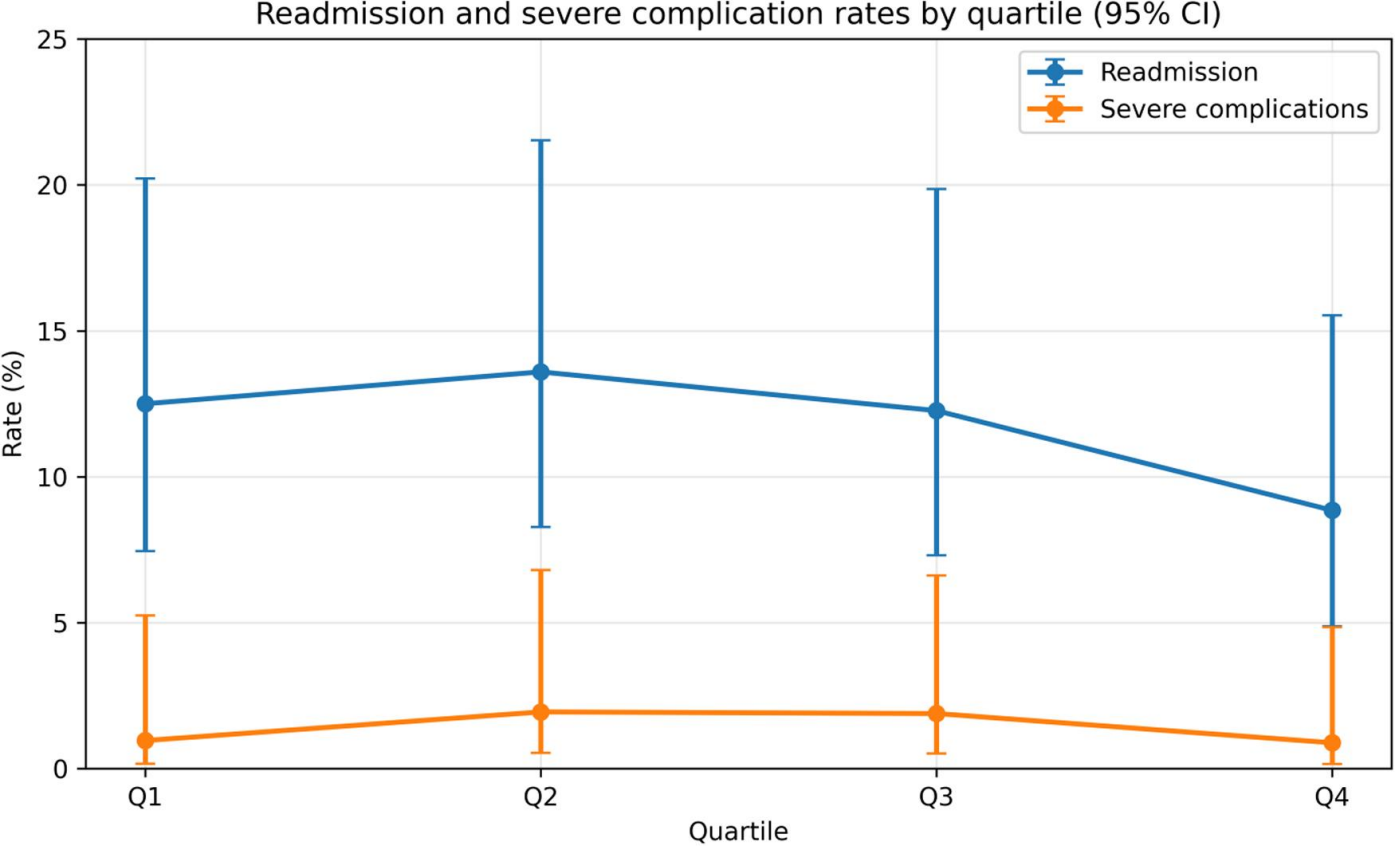
Evolution by quarter of readmission and high-grade complications rate (Q1; *n* = 104; Q2; *n* = 103; Q3; *n* = 106; Q4; *n* = 113).

Evolution over time is illustrated in Figure 2.

In Table 4, data from VISUCHIR are displayed. Our centre showed higher SDD rates (*p* = 0.004) and shorter inpatient stays than national benchmarks, accompanied by more favorable evolution of the 30-day readmission rate (*p* < 0.001).

**Table 4:** VISUCHIR benchmarking indicators comparing national French surgical activity in oncologic surgery with our center implementing the betty coaching digital pathway.

Outcomes	France	Our center
Same-day discharge rate	48.3%	54.2%
Inpatient hospital stay (days)	7.0	4.4
30-day readmission rate evolution (5-year: 2020–2024)	−6.0%	−20.4%
Caseload	440,068	2,678

## Discussion

The present study evaluated the impact of implementing a comprehensive digital perioperative pathway integrating structured prehabilitation, patient education, rehabilitation guidance, on short-term outcomes after elective pelvic gynecological surgery. Although statistical significance was not reached, the observed 34% relative reduction in 90-day readmission rates, along with a decrease in high-grade complications, suggests a numerical signal supporting the value of digitally enabled perioperative optimization. These findings are consistent with the growing body of literature demonstrating that structured perioperative pathways, particularly when enhanced by digital tools, may improve recovery trajectories and reduce unplanned healthcare utilization.

Prehabilitation has emerged over the past decade as a cornerstone of perioperative medicine. Multimodal prehabilitation programs combining physical conditioning, respiratory optimization, nutritional counseling, and psychological preparation have been associated with improved functional capacity and reduced postoperative morbidity in abdominal and oncologic surgery (5–7). However, widespread implementation remains limited by logistical constraints, resource allocation, and patient accessibility barriers (10, 11). Digital platforms provide a scalable alternative by delivering structured content asynchronously while maintaining standardized protocol adherence. In the present cohort, the integration of prehabilitation within a smartphone-based application ensured early initiation following surgical scheduling, likely contributing to enhanced physiologic readiness at the time of surgery. Although functional capacity was not directly measured in this study, prior randomized trials have demonstrated that digital perioperative programs can achieve outcomes comparable to in-person interventions (12).

Beyond physiologic optimization, patient education plays a central role in modulating postoperative recovery. Inadequate understanding of expected postoperative symptoms is a recognized driver of emergency department visits and readmissions (20). Structured digital education may recalibrate patient expectations, improve symptom self-management, and reduce anxiety-driven consultations. The reduction in readmission observed in this study, although not statistically significant, aligns with this hypothesis. Importantly, the readmission rate during the three preimplementation quarters remained stable, suggesting that the decline observed in the fourth quarter was unlikely attributable to temporal variation alone. While residual confounding cannot be excluded, the absence of changes in surgical technique, case mix, or perioperative protocols during the study period strengthens the plausibility of a causal association.

Remote monitoring through electronic patient-reported outcomes constitutes another key mechanism by which digital platforms may influence postoperative outcomes (14). Early identification of symptom deterioration enables timely intervention, potentially preventing progression to severe complications requiring hospitalization. Several prospective studies have demonstrated that structured ePRO monitoring reduces complication severity and healthcare utilization in oncologic and abdominal surgery (12, 17, 21). The reduction from 1.6% to 0.9% in severe complications, while modest in absolute terms, represents a clinically relevant improvement in a population undergoing predominantly minimally invasive procedures with already low baseline morbidity.

Length of stay during the index hospitalization remained stable at 0.86 days, with a same-day discharge rate exceeding 50%. This stability is expected given that institutional enhanced recovery protocols were already optimized prior to digital implementation. In high-performing centers with established ERAS pathways, additional gains in index hospitalization metrics may be limited, and the incremental benefit of digital tools may instead manifest in postdischarge outcomes. This pattern is consistent with the concept that digital health interventions primarily enhance the continuity of care beyond hospital discharge, where traditional monitoring is minimal. The slight reduction in readmission length of stay in the postimplementation cohort further supports the hypothesis that earlier recognition of complications may attenuate severity at presentation.

The interpretation of readmission trends should nevertheless remain cautious given the nonrandomized pre-post design. Readmissions after pelvic gynecological surgery are multifactorial and may be influenced by patient expectations, symptom perception, access to postoperative advice, and early complication recognition. We hypothesize that the BETTY pathway may have contributed to reducing avoidable hospital returns through improved perioperative education, symptom reassurance, rehabilitation guidance, and earlier management of postoperative symptoms. In addition, the reduction in length of stay during readmission episodes (3.2 vs. 2.1 days) may suggest earlier identification and management of postoperative events before progression to more severe clinical presentations.

The nonrandomized pre-post design represents a methodological limitation inherent to real-world implementation studies. However, several factors mitigate this concern. First, consecutive patient inclusion minimized selection bias. Second, surgical procedures were performed by a single experienced surgeon, reducing performance variability. Third, the case mix and procedural complexity remained comparable across quarters. Fourth, outcome capture relied on a national administrative registry (PMSI), ensuring comprehensive hospitalization tracking and reducing attrition bias.

Another consideration relates to digital literacy and engagement. Successful implementation of mobile health interventions depends on patient adherence to application use (15, 22). Although uptake was high in the postimplementation quarter, the study did not formally assess engagement metrics such as completion rates of questionnaires or time spent within educational modules. Future research should incorporate usability assessments and stratify outcomes according to engagement intensity to better understand dose–response relationships. Additionally, socioeconomic determinants of digital access may influence generalizability, although smartphone penetration in the studied population is high (22).

From a health system perspective, even a non-statistically significant reduction in readmission may translate into meaningful cost savings and resource optimization when applied at scale (18). Unplanned readmissions after gynecological surgery contribute to emergency department overcrowding and financial burden. Digital perioperative pathways require upfront technological investment but may offer favorable cost-effectiveness profiles if reductions in complications and readmissions are sustained. Formal economic analyses are warranted to evaluate incremental cost-effectiveness ratios and long-term value.

Postoperative complications were assessed using the institutional Severity Index (SI), an algorithm derived from the French PMSI national coding system integrating comorbidities, procedural complexity, postoperative complications, and length of stay. We acknowledge that the Clavien–Dindo classification is more widely used and internationally recognized for reporting surgical morbidity. Thus, the use of a PMSI-derived institutional severity classification rather than a standardized Clavien–Dindo grading system represents a methodological limitation. However, previous studies have found a high concordance between SI and the Clavien–Dindo classification suggesting the reliability of the SI (19). When looking at patient data needing prolonged hospital stay and/or readmission the rate of high grade complication using Clavien-Dindo classification was 1.9% (95%CI: 0.5–6.7), 2.9% (95%CI: 1.0–8.2), 2.8% (95%CI: 1.0–8.0), and 0.9% (95%CI:0.2–4.8), in Q1, Q2, Q3, and Q4 patients, respectively.

The present study has several limitations that should be acknowledged. First, its retrospective pre-post design inherently limits causal inference and exposes the analysis to potential temporal and selection biases. Although surgical techniques, perioperative protocols, discharge criteria, and the operating surgeon remained unchanged during the study period, unmeasured secular trends or organizational factors may still have influenced postoperative outcomes independently of the digital intervention. Consequently, the observed associations should be interpreted cautiously and considered hypothesis-generating rather than definitive evidence of efficacy. Second, the relatively limited sample size, particularly in the postimplementation cohort, reduced the statistical power to detect significant differences for outcomes with low event rates such as high-grade complications. This limitation likely contributed to the absence of statistical significance despite numerical relative reductions in readmission and severe complications. Third, the study did not formally evaluate patient engagement or adherence to the digital pathway. Metrics such as frequency of application use, completion rates of educational modules and questionnaires, duration of interaction, or response rates to ePRO monitoring were not prospectively collected in that study. However, previous studies demonstrated a high adherence rate (17). Finally, one co-author has ownership interests in AIMED2, the developer of the BETTY platform. Although study outcomes were extracted from an independent national administrative database and analyzed retrospectively, this relationship should be acknowledged as a potential source of bias in study interpretation. Importantly, no *a priori* power calculation was performed. Detecting the observed absolute difference (12.8%–8.8%) with 80% power at *α* = 0.05 would have required approximately 350 patients per arm. Thus, the study was underpowered to assess this endpoint, and has to be considered as hypothesis-generating for further prospective studies.

Despite these limitations, the study also presents several important strengths. Consecutive patient inclusion minimized selection bias and reflects real-world clinical practice. Outcome assessment relied on the PMSI national administrative registry, ensuring comprehensive capture of hospitalization events and reducing attrition or reporting bias. The implementation setting also represents a relevant strength, as the intervention was evaluated within routine practice rather than under highly controlled experimental conditions.

In conclusion, in this retrospective observational pre-post implementation study, introduction of a digital perioperative pathway was associated with favorable postoperative trends, including lower readmission and high-grade complication rates following pelvic gynecological surgery. Given the nonrandomized design and limited sample size, these findings should be considered exploratory and hypothesis-generating. Prospective multicenter randomized studies are required to validate these observations and better define the clinical and economic impact of digital perioperative care pathways.

## Conclusions

In conclusion, the implementation of a comprehensive digital perioperative pathway integrating structured education, prehabilitation guidance, rehabilitation support, was associated with favorable postoperative trends following elective pelvic gynecological surgery. In particular, lower readmission and high-grade complication rates were observed after implementation of the BETTY pathway within an already optimized ERAS-based surgical environment. Given the retrospective pre-post observational design, these findings should not be interpreted as demonstrating a definitive causal effect of the intervention. Rather, they provide exploratory real-world evidence supporting the potential contribution of digitally enabled perioperative care to postoperative recovery and continuity of care after discharge. Further prospective multicenter randomized studies incorporating patient-reported outcomes, functional recovery metrics, engagement analyses, and cost-effectiveness evaluations are required to validate these observations and better define the role of digital perioperative pathways in contemporary gynecological surgery.

## Data Availability

The raw data supporting the conclusions of this article will be made available by the authors, without undue reservation.
