# Supplementary material for: Impact of a digital pre- and rehabilitation program on postoperative outcomes after pelvic gynecological surgery
Source: Front Digit Health. 2026 Jun 26;8:1867518. doi: 10.3389/fdgth.2026.1867518 (PMC13350053; doi:10.3389/fdgth.2026.1867518)
Supplement: Supplementary file 1 [file Table1.docx]

Study design: Retrospective single-center pre-post observational cohort study

| **STROBE Item** | **Recommendation** | **Addressed in Manuscript** |
| --- | --- | --- |
| **Title and Abstract** |  |  |
| 1a | Indicate the study design in the title or abstract | Yes – retrospective pre-post observational study described in Abstract and Methods |
| 1b | Provide balanced summary of methods and findings | Yes |
| **Introduction** |  |  |
| 2 | Explain scientific background and rationale | Yes – Introduction |
| 3 | State specific objectives and hypotheses | Yes – final paragraph of Introduction |
| **Methods** |  |  |
| 4 | Present key elements of study design early in manuscript | Yes – “retrospective single-center pre-post implementation cohort study” |
| 5 | Describe setting, locations, and relevant dates | Yes – tertiary referral center; Feb 2024–Jan 2025 |
| 6a | Describe eligibility criteria and participant selection | Yes – Inclusion and exclusion criteria specified |
| 6b | Describe follow-up methods | Yes – PMSI registry with 90-day follow-up |
| 7 | Clearly define outcomes, exposures, predictors, confounders | Partially – readmission, LOS, complications defined; residual confounding acknowledged |
| 8 | Describe data sources and assessment methods | Yes – PMSI registry and VISUCHIR benchmarking database |
| 9 | Describe efforts to address potential bias | Yes – standardized perioperative management, consecutive inclusion, single surgeon |
| 10 | Explain study size determination | Yes – exploratory study; no a priori sample size calculation |
| 11 | Explain handling of quantitative variables | Yes – continuous and categorical variables specified |
| 12a | Describe statistical methods | Yes – Chi-square, Fisher exact, t-test, Mann–Whitney |
| 12b | Describe subgroup or adjusted analyses | No adjusted analyses performed |
| 12c | Explain handling of missing data | Limited missing data due to registry-based extraction |
| 12d | Address loss to follow-up | Not applicable – registry-based capture |
| 12e | Describe sensitivity analyses | Not performed |
| **Results** |  |  |
| 13a | Report participant numbers at each stage | Yes – 426 included patients |
| 13b | Provide reasons for exclusions | Yes |
| 13c | Consider flow diagram | Yes - Added |
| 14a | Describe participant characteristics | Yes – Table 1 |
| 14b | Indicate missing data | Minimal; registry-based dataset |
| 15 | Report outcome events and summary measures | Yes |
| 16a | Provide unadjusted estimates and precision | Yes – p-values reported |
| 16b | Report category boundaries where relevant | Yes |
| 16c | Translate relative into absolute risks when relevant | Partially – relative reduction described |
| 17 | Report additional analyses | Yes – temporal quarter analysis and VISUCHIR benchmarking |
| **Discussion** |  |  |
| 18 | Summarize key findings with reference to objectives | Yes |
| 19 | Discuss limitations and potential bias | Yes – retrospective design, confounding, sample size |
| 20 | Provide cautious interpretation of findings | Yes – exploratory and hypothesis-generating nature emphasized |
| 21 | Discuss generalizability | Yes – single-center expert setting acknowledged |
| **Other Information** |  |  |
| 22 | Report funding source and role of funders | Yes – Funding statement included |
